# Kynurenic Acid Inhibits the Electrical Stimulation Induced Elevated Pituitary Adenylate Cyclase-Activating Polypeptide Expression in the TNC

**DOI:** 10.3389/fneur.2017.00745

**Published:** 2018-01-16

**Authors:** Tamás Körtési, Bernadett Tuka, János Tajti, Teréz Bagoly, Ferenc Fülöp, Zsuzsanna Helyes, László Vécsei

**Affiliations:** ^1^Faculty of Medicine, Department of Neurology, Albert Szent-Györgyi Clinical Center, University of Szeged, Szeged, Hungary; ^2^MTA-SZTE Neuroscience Research Group, University of Szeged, Szeged, Hungary; ^3^Faculty of Medicine, Department of Pharmacology and Pharmacotherapy, University of Pécs, Pécs, Hungary; ^4^Faculty of Pharmacy, Institute of Pharmaceutical Chemistry, University of Szeged, Szeged, Hungary; ^5^MTA-SZTE Stereochemistry Research Group, Hungarian Academy of Sciences, Szeged, Hungary; ^6^János Szentágothai Research Center, University of Pécs, Pécs, Hungary; ^7^MTA-PTE NAP B Chronic Pain Research Group, University of Pécs, Pécs, Hungary

**Keywords:** migraine, trigeminovascular system, pituitary adenylate cyclase-activating polypeptide, kynurenic acid, MK-801

## Abstract

**Background:**

Migraine is a primary headache of imprecisely known mechanism, but activation of the trigeminovascular system (TS) appears to be essential during the attack. Intensive research has recently focused on pituitary adenylate cyclase-activating polypeptide (PACAP) and the kynurenine systems as potential pathogenic factors.

**Aim:**

We investigated the link between these important mediators and the effects of kynurenic acid (KYNA) and its synthetic analog (KYNA-a) on PACAP expression in the rat trigeminal nucleus caudalis (TNC) in a TS stimulation model related to migraine mechanisms.

**Methods:**

Adult male Sprague-Dawley rats were pretreated with KYNA, KYNA-a, the NMDA receptor antagonist MK-801, or saline (vehicle). Next, the trigeminal ganglion (TRG) was electrically stimulated, the animals were transcardially perfused following 180 min, and the TNC was removed. In the TNC samples, 38 amino acid form of PACAP (PACAP_1–38_)-like radioimmunoactivity was measured by radioimmunoassay, the relative optical density of preproPACAP was assessed by Western blot analysis, and PACAP_1–38_ mRNA was detected by real-time PCR.

**Results and conclusion:**

Electrical TRG stimulation resulted in significant increases of PACAP_1–38_-LI, preproPACAP, and PACAP_1–38_ mRNA in the TNC. These increases were prevented by the pretreatments with KYNA, KYNA-a, and MK-801. This is the first study to provide evidence for a direct link between PACAP and the kynurenine system during TS activation.

## Introduction

Migraine is a neurovascular primary headache disorder with a complex genetic background (1, 2), the pathomechanism of which is still not fully understood. One of the proposed mechanisms of the disease is the activation and sensitization of the trigeminovascular system (TS). However, central sensitization and hyperexcitability due to excessive glutamate release are also suspected to be involved in the development and persistence of pain.

Calcitonin gene-related peptide (CGRP) is one of the main regulators of the TS, and the recently identified pituitary adenylate cyclase-activating polypeptide (PACAP) also plays key roles (3–6). In migraineurs, the level of CGRP in the peripheral blood is increased during a migraine attack compared with the interictal period (4). A very similar observation has recently been made for PACAP as well, suggesting a potential biomarker function of PACAP in migraine (7). In addition, similarly to CGRP (8), intravenous administration of the 38 amino acid form of PACAP (PACAP_1–38_) provoked headache and vasodilatation, both in healthy participants and migraine sufferers, whereas it delayed migraine-like attacks only in migraineurs (9–11).

In experimental conditions, intraperitoneal administration of PACAP_1–38_ evoked marked photophobia, meningeal vasodilatation and increased the number of c-fos-positive activated neurons in the spinal trigeminal nucleus caudalis (TNC) in wild type, but not in PACAP_1–38_-deficient mice (12). In line with this, an increased concentration of PACAP_1–38_ was detected in the TNC after the activation of the TS in different animal models (13).

Interesting and promising endogenous regulators of glutamatergic neurotransmission include certain metabolites of the kynurenine pathway formed during the catabolism of tryptophan. Some of these metabolites are neuroactive and play essential roles in the modulation of *N*-methyl-d-aspartate glutamate receptor (NMDAR) function. Since glutamate-induced overexcitation plays a significant role in a number of neuronal diseases, the kynurenine system has recently become the subject of intense neurobiological research (14).

Kynurenic acid (KYNA), an endogenous NMDAR antagonist, is one of the end products of this pathway, which can dose-dependently inhibit the activation and sensitization of TS (15). However, the penetration of KYNA across the blood–brain barrier is very limited, necessitating the development of synthetic analogs with potentially better pharmacokinetic properties to exploit its therapeutic potential. In a chemically induced animal model of migraine, a derivative of KYNA was able to inhibit nitroglycerin (NTG)-induced increase of c-fos (16) and CGRP (17) expression in rat TNC. Furthermore, the same KYNA analog promoted an anti-inflammatory response in the trigeminal ganglion (TRG) in a complete Freund’s adjuvant (CFA)-induced model of dural neuroinflammation (18).

To date, only a few studies have provided evidence for an association between PACAP and the NMDAR. Calcium influx through the NMDAR is an important mediator of PACAP expression, inducing gene transcription *via* the mitogen-activated protein kinase signaling cascade (19). PACAP has been demonstrated to enhance the functional coupling of neuronal nitrogen-monoxide synthase and the NMDAR in models of both inflammatory and neuropathic pain (20).

Based on all these recent findings, the aim of this study was to investigate a potential association between the KYNA system and PACAP expression, with particular regard to NMDAR activation and a possible novel therapeutic function for the stable KYNA analog in terms of its ability to influence migraine-related elevation of PACAP levels.

## Materials and Methods

### Animals

The study used 76 young adult (10–12 weeks old) male Sprague-Dawley rats for the experiments; 18 intact non-stimulated animals in the control group and a total of 58 animals in the groups undergoing electrical stimulation of the TRG (ES-TRG) to model TS activation in migraine. The animals were bred and maintained under standard laboratory conditions on a 12- to 12-h light cycle at 24 ± 1°C and approximately 50% relative humidity in the Laboratory Animal House of the Department of Neurology. The rats had free access to standard rat chow and water.

### Ethics

All experimental procedures performed in this study complied fully with the guidelines of Act 1998/XXVIII of the Hungarian Parliament on Animal Experiments (243/1988) and with the recommendations of the International Association for the Study of Pain and European Communities Council (86/609/ECC). The studies were in harmony with the Ethical Codex of Animal Experiments and were approved by the Ethics Committee of the Faculty of Medicine, University of Szeged, XXIV./352/2012.

### Drugs

MK-801 and KYNA were obtained from Sigma-Aldrich Corporation (St. Louis, MO, USA). The analog of KYNA (KYNA-a) was synthesized by Prof. Fülöp Ferenc and his research team in the Department of Pharmaceutical Chemistry, University of Szeged. KYNA was administered at 189.17 mg/kg body weight, MK-801 at 4 mg/kg body weight, and KYNA-a at 295.76 mg/kg body weight dose. The substances were dissolved in 1.5 ml of physiological saline solution, and pH was adjusted with sodium hydroxide and hydrogen chloride.

### Experimental Protocol

**Figure 1:**
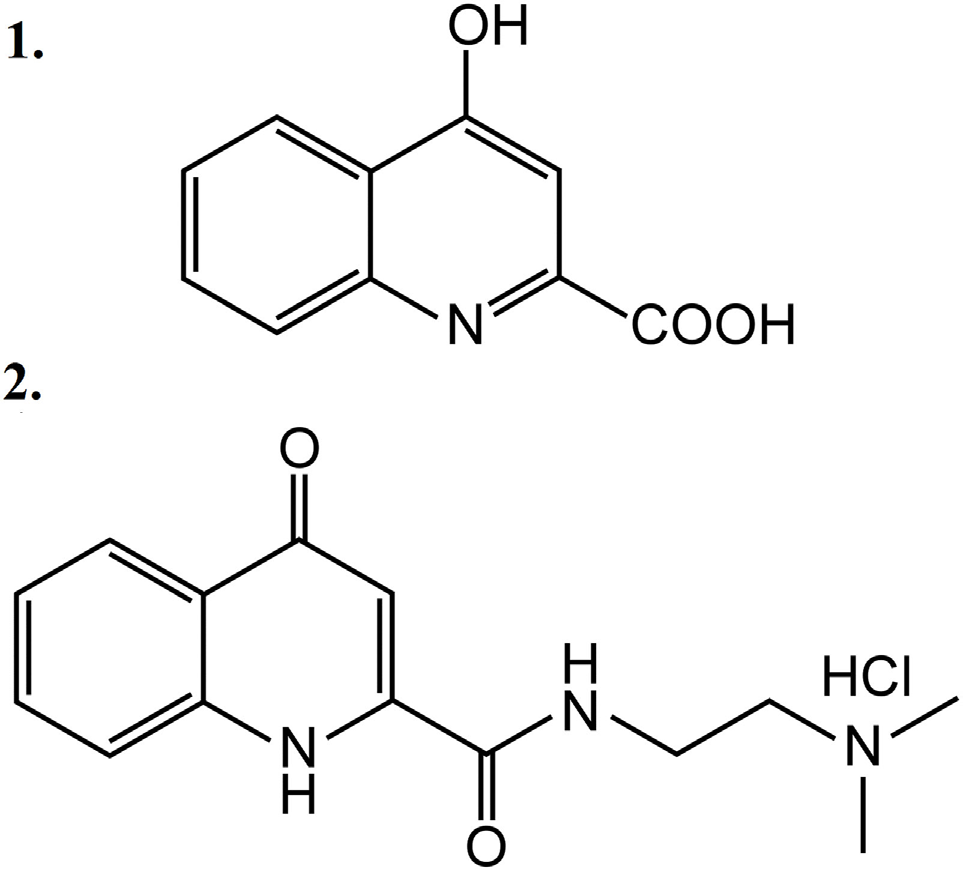
Structural formulae of kynurenic acid **(1)** and kynurenic acid synthetic analog **(2)**.

Five animal groups were created: one non-stimulated control group and four ES-TRG groups with different pretreatments. The group pretreated with vehicle (physiological saline i.v.) was used as the negative control, whereas that pretreated with MK-801 (a known selective NMDAR antagonist) served as the positive ES-TRG control. Two additional groups were pretreated with KYNA and KYNA-a, respectively. The structural formulae of KYNA and KYNA-a are presented in Figure 1. All drugs were dissolved in 1.5 ml physiological saline. After optimization of pH, the solution was supplemented with physiological saline to a total volume of 2 ml.

First, the rats were anesthetized with intraperitoneal 4% chloral hydrate solution (10 ml/kg bw dose), and the anesthesia was maintained throughout the experiment. After intravenous administration of the pretreatments, the animals were placed in a stereotaxic setup. The head was fixed and the right TRG was localized. A stimulating electrode (FHC concentric bipolar electrode, OP: 200 µm SS; IP: 50 µm Pt/lr) was introduced into the TRG, and electrical stimulation (ES) was performed according to the following parameters: duration: 30 min; stimulation rate: 10 Hz; current: 1 mA; stimulation mode: continuous. Results of our previous studies suggested that the concentration of PACAP_1–38_ in the central nervous system is highest 3 h after the ES-TRG; therefore, transcardial perfusion of the animals with 200 ml phosphate-buffered saline was initiated 180 min after stimulation, followed by the dissection of the TNC (13). In the non-stimulated group, perfusion and dissection were carried out following anesthesia. After preparation, the samples were stored at −80°C until further use. The concentration of PACAP_1–38_ was measured by radioimmunoassay (RIA), the relative optical density of preproPACAP was analyzed by Western blot, and the relative gene expression of PACAP_1–38_ was determined by real-time polymerase chain reaction (RT-PCR). The analytical methods and their respective subject numbers are presented in Table 1.

**Table 1:** The applied analytical methods and their respective subject number.

Radioimmunoassay			Western blot analysis			Real-time PCR		
Treatment	Electrical stimulation of the TRG (ES-TRG)	*n*	Treatment	ES-TRG	*n*	Treatment	ES-TRG	*n*
–	–	7	–	–	6	–	–	5
0.9% NaCl	+	7	0.9% NaCl	+	6	0.9% NaCl	+	5
Kynurenic acid synthetic analog	+	7	Kynurenic acid (KYNA)	+	6	KYNA	+	5
–			KYNA-a	+	6	KYNA-a	+	5
–			MK-801	+	6	MK-801	+	5

### Radioimmunoassay

The frozen samples were weighed and homogenized in 1 ml ice-cold double-distilled water with a manual Potter homogenizer. The homogenates were centrifuged at 10,000 rpm for 10 min and then at 12,000 rpm for an additional 10 min, and 25 µl samples of the supernatants were used for RIA measurements. The tracer was mono-^125^I-labeled PACAP_1–38_ prepared in our laboratory. The synthetic peptide was used as the RIA standard in concentrations ranging from 0 to 2,000 fmol/ml. The assay buffer was composed of 0.05 M (pH 7.4) phosphate buffer containing 0.1 M sodium chloride, 0.25% (w/v) bovine serum albumin (BSA), and 0.05% (w/v) sodium azide. The anti-PACAP_1–38_ (Abcam, ab35342; Lot. No.: GR48676-4; 100 µl, 1:10,000 dilution), the RIA tracer (100 µl, 3,000 cpm/tube), and the standards or samples of unknown concentration (100 µl) were injected into polypropylene tubes containing 700 µl assay buffer (1 ml total volume). After incubation for 48–72 h at 4°C, the antibody-bound peptide was separated from the free peptide by the addition of 100 µl separating solution (10 g charcoal, 1 g dextran, and 0.5 g commercial nonfat milk powder dissolved in 100 ml distilled water). Following centrifugation at 3,000 rpm at 4°C for 20 min, the contents of the tubes were gently decanted, and the radioactivity of the precipitates was measured in a gamma counter (gamma, type: NZ310). PACAP_1–38_-like immunoreactivities of the samples were calculated from the calibration curve and expressed as fentomoles per milligram wet tissue (7, 13).

### Western Blot Analysis

The samples were sonicated in ice-cold buffer containing 50 mM Tris–HCl, 150 mM NaCl, 0.1% Igepal, 0.1% cholic acid, 2 mg/ml leupeptin, 2 mM phenylmethylsulphonyl fluoride, 1 mg/ml pepstatin, 2 mM ethylenediaminetetraacetic acid, and 0.1% sodium dodecyl sulfate (SDS). After homogenization, the samples were centrifuged at 12,000 rpm for 10 min at 4°C, and supernatants were aliquoted and stored at 20°C until use. The protein concentration was determined by the BCA Protein Assay Kit using BSA as the standard. Before protein separation, each sample was mixed with the sample buffer and denatured by boiling for 5 min. Equal amounts of protein samples (20 mg/lane) were separated by standard SDS polyacrylamide gel electrophoresis on 10% Tris–Glycine gel and electrotransferred onto an AmershamHybond-ECL nitrocellulose membrane (0.45-mm pore size). We used the Page Ruler Prestained Protein Ladder (10–170 kDa) to define approximate molecular weights. Following blotting, the membranes were blocked for 1 h at room temperature in Tris-buffered saline containing Tween 20 (TBST) and 5% nonfat dry milk powder. Subsequently, the membranes were incubated in TBST containing 1% nonfat dry milk and anti-PACAP antibody (against the C terminal; ab174982, dilution: 1:500, incubation parameters: overnight, 4°C), anti-glyceraldehyde 3-phosphate dehydrogenase (GAPDH) antibody (D16H11, dilution: 1:1,000, incubation parameters: overnight, room temperature). The following day, the membranes were incubated in TBST containing 1% nonfat dry milk powder and horseradish peroxidase-conjugated goat anti-rabbit secondary antibody (sc-2030, Santa Cruz Biotechnology) for 2 h at room temperature. The protein bands were revealed with SuperSignal West Pico Chemiluminescent Substrate using Carestream Kodak BioMax Light film.

### Real-time PCR

For RT-PCR analysis, total RNA was isolated from the TNC with Trizol, according to the manufacturer’s protocol. RNA concentrations were determined using a MaestroNano spectrophotometer, and the verification of RNA integrity was performed by gel electrophoresis, using 1% agarose gel. cDNA was synthesized from 1 µg total RNA with random hexamer primers by RevertAid First Strand cDNA Synthesis Kit (Thermo Scientific, USA). The produced cDNA was stored at −20°C until further use. The RT-PCR was performed on a CFX 96 Real-Time System (Bio-Rad, USA) to detect changes in mRNA expression, using forward (5′-CCTACCGCAAAGTCTTGGAC-3′) and reverse (5′-TTGACAGCCATTTGTTTTCG-3′) primers designed for PACAP_1–38_. Thermal cycling conditions were 95°C for 2 min followed by 40 cycles of 95°C for 10 s and 60°C 30 s. The relative mRNA level was calculated by the 2^−ΔΔCt^ method. A pre-optimized primer and probe assay for 18S rRNA was used as an endogenous control (Applied Biosystems, USA).

### Statistical Analysis

The Shapiro–Wilk test was used to determine the distribution of data. It shows that our data do not follow the normal distribution so after the Kruskal–Wallis test we used the Tukey *post hoc* test to analyze the results. A probability level of *p* < 0.05 was considered significant. Median ± interquartile ranges were represented in the box-plot figures.

## Results

### Electrical TRG-Stimulation-Induced PACAP_1–38_-LI Increase in the TNC Is Prevented by KYNA-a Pretreatment

**Figure 2:**
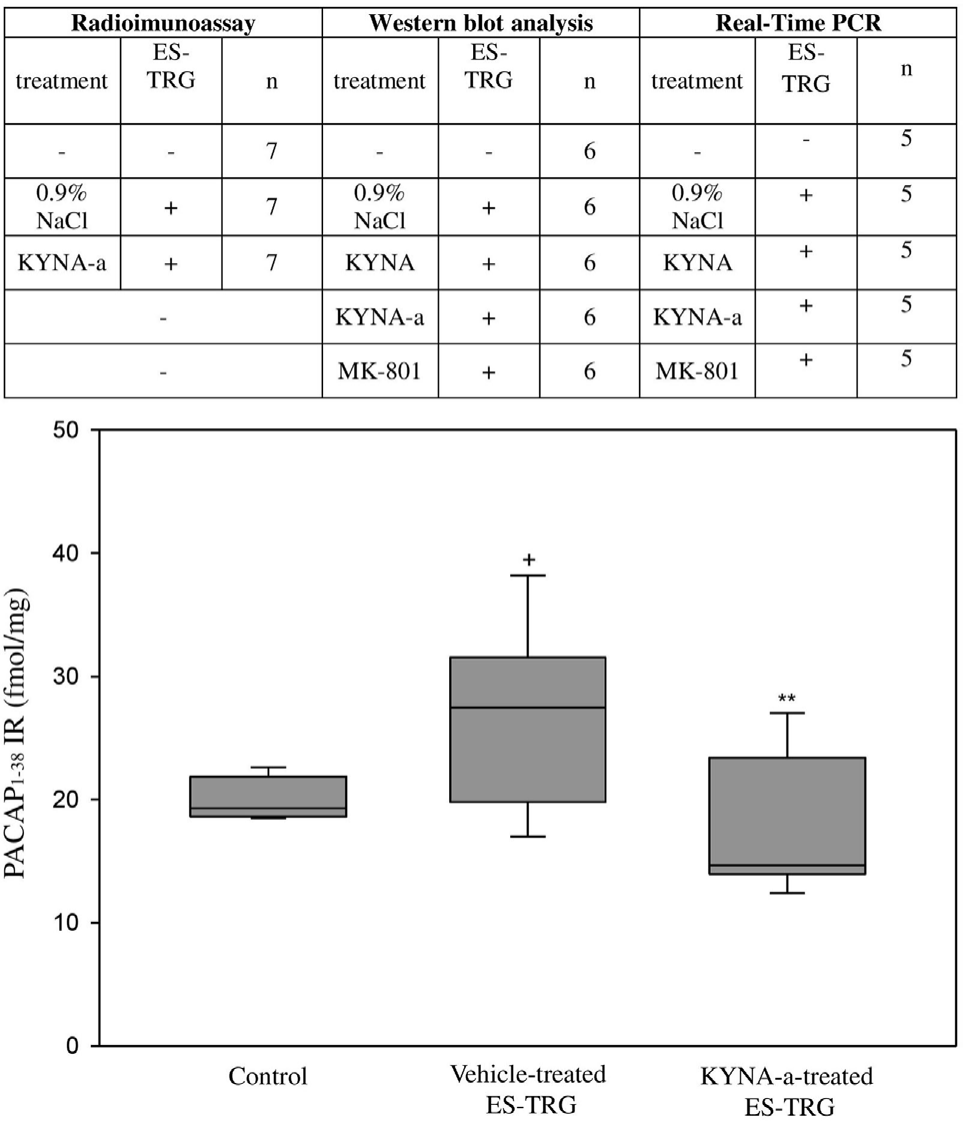
Immunoreactivity of 38 amino acid form of PACAP (PACAP_1–38_) in the TNC following electrical stimulation of the trigeminal ganglion. ^+^*p* < 0.001 vs. Control group, **p < 0.005 vs. Vehicle-treated electrical stimulation of the TRG (ES-TRG) group. Black lines demonstrate medians. Median ± interquartile range (*n* = 7).

Electrical stimulation of the TRG resulted in a significant increase of PACAP_1–38_-LI in the TNC of the vehicle-treated group (27.49 fmol/mg) compared with the intact non-stimulated control group (19.31 fmol/mg; *p* < 0.05). KYNA-a-pretreatment prevented this ES-TRG-evoked elevation (14.68 fmol/mg) when compared with the stimulated, vehicle-treated animals (*p* < 0.01) (Figure 2).

### Electrical TRG-Stimulation-Induced preproPACAP Increase in the TNC Is Prevented by KYNA-a, KYNA, and MK-801 Pretreatment

**Figure 3:**
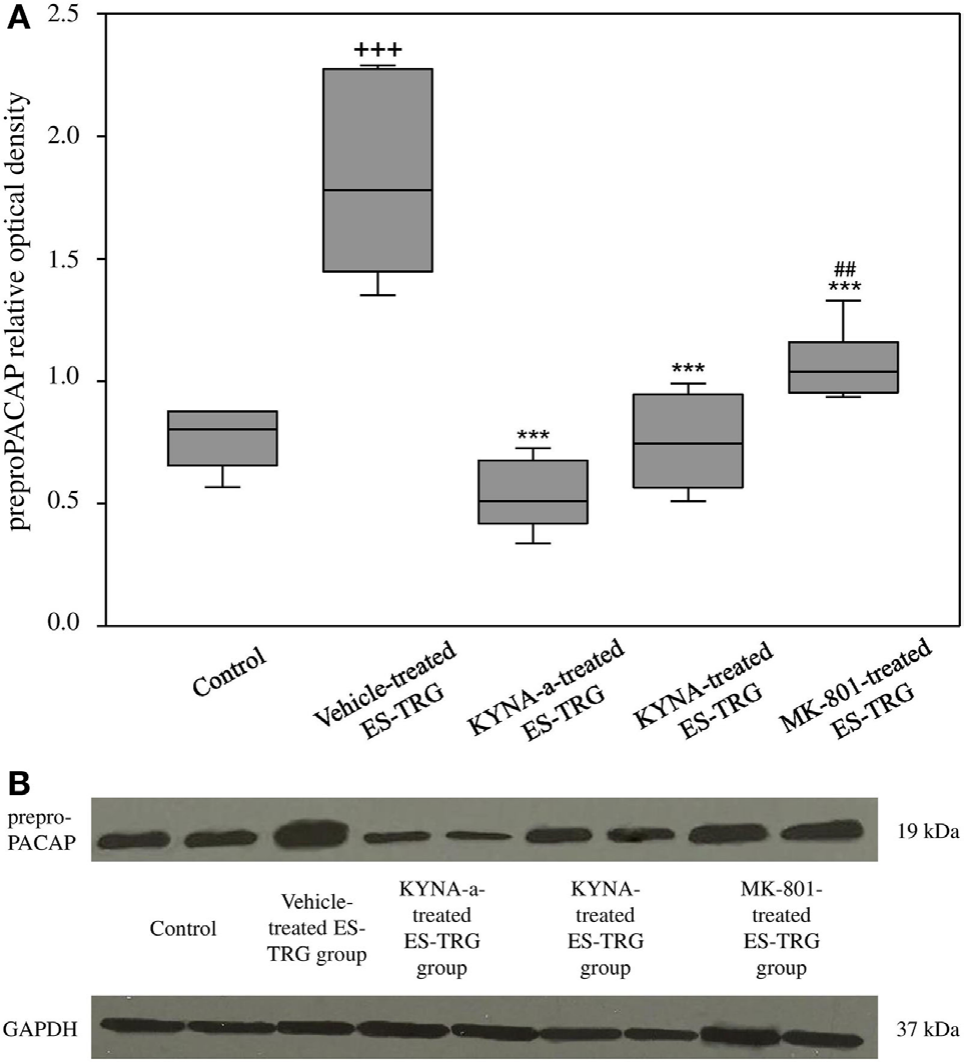
**(A)** Relative optical density of the preproPACAP protein in the TNC following electrical stimulation of the trigeminal ganglion. ^+++^*p* < 0.001 vs. Control group. ***p < 0.001 vs. Vehicle-treated electrical stimulation of the TRG (ES-TRG) group. ^##^*p* < 0.01 vs. kynurenic acid synthetic analog (KYNA-a)-treated ES-TRG group. Black lines demonstrate medians. Median ± interquartile range (*n* = 6). **(B)** Western blot of preproPACAP and glyceraldehyde 3-phosphate dehydrogenase (GAPDH) expression in the TNC.

A significant increase in the relative optical density of preproPACAP protein was observed in the vehicle-treated ES-TRG group (1.78) as compared with the non-stimulated control group (0.81; *p* < 0.001). The relative optical density values of preproPACAP in the ES-TRG groups pretreated with KYNA (0.74), KYNA-a (0.51), or MK-801 (1.04) were significantly reduced compared with the vehicle-treated ES-TRG group (*p* < 0.001). The difference of relative optical density between the MK-801- and the KYNA-a-treated groups was significant (*p* < 0.01). No significant differences were observed between the KYNA- and KYNA-a-treated groups. No differences were found with either the KYNA or MK-801-treated groups (Figures 3A,B).

### Electrical TRG-Stimulation-Induced Increased PACAP_1–38_ mRNA Expression in the TNC Is Prevented by KYNA-a, KYNA, and MK-801 Pretreatment

**Figure 4:**
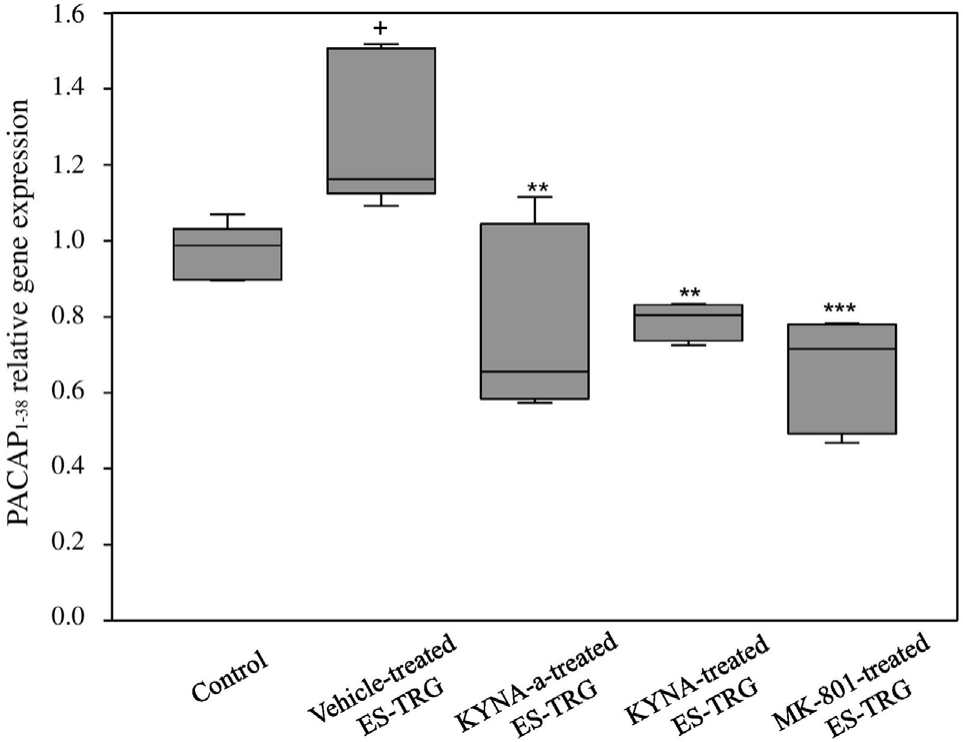
Relative mRNA expression of 38 amino acid form of PACAP (PACAP_1–38_) in the TNC following electrical stimulation of the trigeminal ganglion. ^+^*p* < 0.05 vs. Control group. **p < 0.01 vs. Vehicle-treated electrical stimulation of the TRG (ES-TRG) group. ***p < 0.001 vs. Vehicle-treated ES-TRG group. Black lines demonstrate medians. Median ± interquartile range (*n* = 5).

A significant increase in the relative mRNA expression of PACAP_1–38_ was observed in the vehicle-treated ES-TRG group (1.162) when compared with the non-stimulated control group (0.987; *p* < 0.05). The relative mRNA expression of PACAP_1–38_ in the ES-TRG groups pretreated with KYNA (0.804), KYNA-a (0.655; *p* < 0.01), or MK-801 (0.715) were significantly reduced compared with the vehicle-treated ES-TRG group (*p* < 0.001). Comparison of the different pharmacology-treated groups did not reveal any significant difference (Figure 4).

## Discussion

In recent years, a number of clinical studies investigated the possible relevance of PACAP in migraine. In patients suffering from migraines without aura, the development of PACAP_1–38_-evoked migraine-like attack was independent of the severity of family load (21). In the same study, 90 min after the injection, the levels of numerous markers relevant in the disease (such as vasoactive intestinal polypeptide, prolactin, S100B, and thyroid-stimulating hormone) were increased in the plasma (22). In addition, magnetic resonance imaging angiography examinations revealed that PACAP_1–38_-induced headache was associated with prolonged vasodilatation of the middle meningeal artery (MMA), but not the middle cerebral artery (MCA) (23). The serotonin 5-HT1B/D receptor agonist, sumatriptan, was able to alleviate the headache, which mirrored the contraction of the MMA, but not the MCA, suggesting that PACAP_1–38_-induced headaches may arise from the extracerebral arteries (23). These human and experimental data suggest that PACAP may be a key molecule in the pathomechanism of migraine.

Several lines of evidence reflect that abnormality of the kynurenine pathway plays roles in several diseases (24). Results of animal experiments suggest a link between the kynurenine pathway and migraine. Indeed, several studies have demonstrated that the NMDAR inhibitor KYNA and its analogs have antinociceptive effects at the levels of both first- and second-order sensory neurons (25). In the animal model of CFA-induced TS activation, KYNA and one of its derivatives both decreased the levels of several inflammatory mediators in the TRG (26). Recently, the effects of two KYNA analogs have been tested in the orofacial formalin model, revealing that the two analogs were able to inhibit the formalin-induced behavioral and morphological changes, and increased the concentration of KYNA in the rat TNC (27). Notably, systemic administration of NTG decreased the expression of kynurenine aminotransferase II in the TS of rats, an enzyme catalyzing the transformation of l-kynurenine to KYNA (28). In line with this, in another model of TS activation, decreased KAT immunoreactivity was observed in mast cells, Schwann cells, and dural macrophages (29). Besides animal experimental data, clinical results have also provided evidence for the connection between the kynurenine system and various headache disorders, including migraine. Indeed, in patients suffering from cluster headache or chronic migraine, alterations of the kynurenine pathway were observed, which among others manifested in the reduction of KYNA concentration in the serum (30).

**Figure 5:**
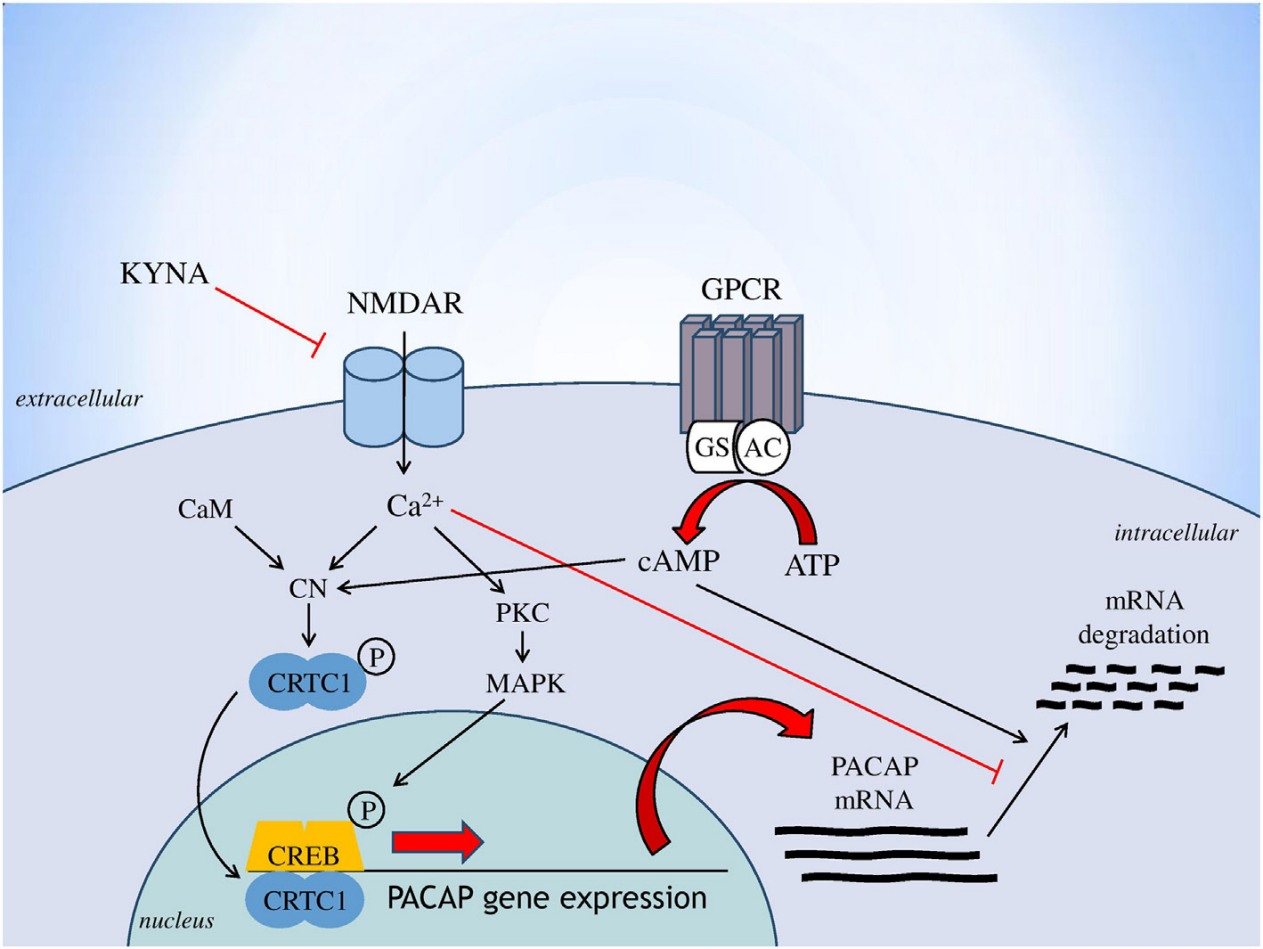
Regulation of PACAP gene expression [based on Fukuchi et al. (19), genes to cells]. Abbreviations: AC, adenylate cylase; ATP, adenosine monophosphate; CaM, calmodulin; cAMP, cyclic adenosine monophosphate; CN, calcineurin; CREB, cAMP response element binding protein; CRTC1, CN/Cre binding protein; GPCR, G protein-coupled receptor; Gs, stimulatory G protein; KYNA, kynurenic acid; MAPK, mitogen-activated protein kinase; NMDAR, *N*-methyl-d-aspartate glutamate receptor; PKC, protein kinase C; PACAP, pituitary adenylate cyclase-activating polypeptide.

Based on the consistent data linking glutamatergic transmission (especially through NMDARs), the kynurenine pathway and PACAP to the pathogenesis of migraine, this is the first study that investigated the relationship between these systems. Findings that all proposed antagonists of the NMDAR were used here (KYNA, KYNA-a and the reference compound MK-801) decreased the expression of PACAP, both at the levels of proteome and transcriptome. One of innovation of this study, that KYNA-a can downregulate both protein and gene expression of preproPACAP and PACAP_1–38_, suggesting that KYNA-a is a potential new drug in the PACAP-targeted migraine therapy in the future. However, the excessive reduction of PACAP expression level should be considered. It is crucial and hard to find the optimal concentration of this peptide, because shifting in the PACAP balance can lead to several symptoms (protective vs. harmful effects) due to the pleiotropic nature of this peptide. To reveal the long-term effects of PACAP in the migraine is needs further investigations. Our results are consistent with previous studies, which reported that the main mediator of PACAP gene expression is intracellular calcium homeostasis (19). Indeed, besides the action of voltage-dependent calcium channels, the main inducer of PACAP gene expression is calcium influx through the NMDARs. The drugs used in our experiment block the NMDAR, thereby moderating the amount of calcium coming into the cell, which may result in decreased PACAP gene expression. The proposed mechanism for the regulation of PACAP gene expression is presented in Figure 5. The other interesting observation of this study that the expression levels of preproPACAP are significantly different between the MK-801- and the KYNA-a-treated groups, raises the possibility of the involvement of additional KYNA targets besides NMDA. Our opinion is that the differences between the variant drug-treated groups are based on the receptor specificity of the molecules. There is evidence that besides NMDAR, KYNA has an effect on the AMPA, kainate, aryl hydrocarbon, G protein-coupled receptor 35, and opiate receptor (31). Regarding KYNA-a, investigations are in process, but the exact targets and mechanisms of this analog have not yet been identified. Presumably, KYNA-a has similar targets to the base compound. Our findings raise the role of these receptors in migraine and draw attention to the importance of further examinations (Figure 5).

In other studies, associations between these systems have also been revealed. Immunohistochemical data suggest a possible interaction between glutamatergic, PACAP, and CGRP systems, based on the expression pattern of PACAP and glutamate in the TRG (32). In addition, a study investigating contextual fear conditioning in rats reported that PACAP participated in the consolidation and extinction *via* NMDARs (33).

Our results provided the direct evidence that NMDAR inhibition can prevent the overexpression of PACAP in an experimental model of migraine and support the idea that therapies aimed at the modulation of glutamatergic transmission, including the use of KYNA derivatives, may be of therapeutic value in migraine. These data contribute to the better understanding of migraine pathogenesis and thereby to the development of more efficient therapeutic approaches.

## Ethics Statement

All experimental procedures performed in this study complied fully with the guidelines of Act 1998/XXVIII of the Hungarian Parliament on Animal Experiments (243/1988) and with the recommendations of the International Association for the Study of Pain and European Communities Council (86/609/ECC). The studies were in harmony with the Ethical Codex of Animal Experiments and were approved by the Ethics Committee of the Faculty of Medicine, University of Szeged, XXIV./352/2012.

## Author Contributions

TK: participated in the design and implementation of experiments, and statistical analysis, interpreted data and wrote the manuscript; BT: participated in the implementation of the experiments and statistical analysis; JT: participated in the conception and design of the experiments, the interpretation of the data, and writing; all the authors: critical revision of the manuscript; TB: participated in RIA measurements; FF: synthesized KYNA-a; ZH: participated in coordinating RIA measurements, in the final approval of the version to be published; LV: participated in the design of the experiments and in the final approval of the version to be published.

## Conflict of Interest Statement

The authors declare that the research was conducted in the absence of any commercial or financial relationships that could be construed as a potential conflict of interest.

## Abbreviations

BSA, bovine serum albumin; CFA, complete Freund’s adjuvant; CGRP, calcitonin gene-related peptide; EDTA, ethylenediaminetetraacetic acid; ES, electrical stimulation; ES-TRG, electrical stimulation of the TRG; IQRs, interquartile ranges; KAT II, kynurenine aminotransferase II; KYNA, kynurenic acid; KYNA-a, kynurenic acid synthetic analog; MAPK, mitogen-activated protein kinase; MCA, middle cerebral artery; MMA, middle meningeal artery; NMDAR, *N*-methyl-d-aspartate glutamate receptor; nNOS, neuronal nitrogen-monoxide synthase; NTG, nitroglycerin; PACAP, pituitary adenylate cyclase-activating polypeptide; PACAP_1–38_, 38 amino acid form of PACAP; PBS, phosphate-buffered saline; RIA, radioimmunoassay; RT-PCR, real-time polymerase chain reaction; SDS, sodium dodecyl sulfate; TBST, Tris-buffered saline containing Tween 20; TNC, trigeminal nucleus caudalis; TRG, trigeminal ganglion; TS, trigeminovascular system; TSH, thyroid-stimulating hormone; VIP, vasoactive intestinal polypeptide.
